# Cellular transfection using rapid decrease in hydrostatic pressure

**DOI:** 10.1038/s41598-024-54463-5

**Published:** 2024-02-26

**Authors:** Shudi Huang, Nan Ji Suo, Tyler R. Henderson, Robert B. Macgregor, Jeffrey T. Henderson

**Affiliations:** 1 Department of Pharmaceutical Sciences, University of Toronto, Toronto, ON M5S 3M2 Canada; 2 Department of Cell and Systems Biology, University of Toronto, Toronto, ON M5S 3G5 Canada; 3 Department of Medical Genetics, Lunenfeld-Tanenbaum Research Institute, Mount Sinai Hospital, Toronto, ON M5G 1X5 Canada

**Keywords:** Biophysical methods, Gene delivery, Transfection, Biological techniques, Stem cells, Embryonic stem cells, Biophysics, Membrane biophysics

## Abstract

Of all methods exercised in modern molecular biology, modification of cellular properties through the introduction or removal of nucleic acids is one of the most fundamental. As such, several methods have arisen to promote this process; these include the condensation of nucleic acids with calcium, polyethylenimine or modified lipids, electroporation, viral production, biolistics, and microinjection. An ideal transfection method would be (1) low cost, (2) exhibit high levels of biological safety, (3) offer improved efficacy over existing methods, (4) lack requirements for ongoing consumables, (5) work efficiently at any scale, (6) work efficiently on cells that are difficult to transfect by other methods, and (7) be capable of utilizing the widest array of existing genetic resources to facilitate its utility in research, biotechnical and clinical settings. To address such issues, we describe here Pressure-jump-poration (PJP), a method using rapid depressurization to transfect even difficult to modify primary cell types such as embryonic stem cells. The results demonstrate that PJP can be used to introduce an array of genetic modifiers in a safe, sterile manner. Finally, PJP-induced transfection in primary versus transformed cells reveals a surprising dichotomy between these classes which may provide further insight into the process of cellular transformation.

## Introduction

Subsequent to Hite's description of the pressure induced inactivation of microorganisms in milk in 1899^1^, the effects of hydrostatic pressure have been extensively studied in both normal and pathologic cells. Industrially, high hydrostatic pressure up to 900 MPa (0.1 MPa = 1 bar = 0.987 atm) has been used to sterilize and preserve thermally sensitive foods without loss of nutritional content or organoleptic properties; a process begun commercially in the 1990s in Japan^2,3^. Clinically, high hydrostatic pressure has also been used to sterilize bone transplants and generate anti-tumor peptides^4–6^. Generally this process involves subjecting samples to a defined pressure for 10–30 min followed by a rapid return to atmospheric pressure, typically within the span of several seconds.

The effects of hydrostatic pressure on biological processes arise from changes induced in the conformational states of proteins, nucleic acids, lipid membranes and their interactions. At pressures below 100 MPa the structure of many proteins are stabilized. Above ~ 200 MPa, tertiary and quaternary structures are frequently altered and above ~ 400 MPa protein denaturation can occur^7–10^. The conformational changes induced by hydrostatic pressure arise from changes in the ionization state of charged amino acids, hydrophobic interactions, amino acid hydration and a loss of the void volumes present in the protein structure due to the imperfect packing of polypeptide chains within the protein interior. Secondary protein structures such as α-helices and β-pleated sheets are quite resistant to pressure, remaining stable at pressures up to 1000 MPa^11^. However, for proteins possessing quaternary structure, subunit interactions may be altered at significantly lower pressures. For example pressures of 50–300 MPa have been shown to alter the equilibrium of microtubules toward their constituent subunits^12–14^; an effect which is rapidly reversed upon return to atmospheric pressure^9,15^. Similarly while the conformational stability of some non-canonical nucleic acid structures such as the G-quadruplex can be destabilized by high pressure, double helical DNA is resistant to pressure-induced conformational changes, up to several hundred MPa^16^.

Of primary cellular constituents, membranes are the most labile to pressure-induced changes^11^. Within the range of 0.1–100 MPa^9,17^, phospholipid bilayers undergo phase transition from a fluid-crystalline state to a more ordered gel-like conformation, with the lipid headgroups being relatively incompressible compared to the bilayer interior^17,18^. As a result of this elevated pressure, the packing density of lipid chains increases leading to enhancement of membrane thickness and a concomitant decrease in the cross-sectional area of the lipid chain^19,20^. Thus with increasing pressure, phospholipid acyl chains become more ordered and the void volumes between lipids decreases, giving rise to the negative volume changes observed for these transitions^21^. These pressure-induced changes can affect cellular functions related to membrane fluidity, such as membrane permeability, ion transport, and signal transduction. An example of this can be seen in NMR studies demonstrating that membrane-associated Ras undergoes significant adaptations between 60–100 MPa with respect to membrane mobility and partitioning^22^. Indeed the high barosensitivity of lipid membranes is thought be a prime factor in determining tolerable pressure limits for specific cell types, with lethality for eukaryotic cells typically observed at pressures between 100–200 MPa as a function of exposure time^18^. Interestingly cancer cells are typically capable of surviving exposure to higher hydrostatic pressures than primary cells which may be attributed to differences in mechanical properties of the cell membrane and cytoskeleton often seen between transformed and healthy counterparts^17^.

Lower hydrostatic pressures, in the range of 30–100 MPa have also been shown to induce alterations in morphology and function in a variety of cell types^13–15^. However changes induced within this pressure range appear reversible upon return to atmospheric pressure. For example, pressure-induced disruptions of mitosis, protein synthesis and the actin cytoskeleton with exposure to ≤ 90 MPa for 10–30 min all demonstrate reversibility upon return to ambient pressure^13,18,23,24^. Thus exposure to pressures of this magnitude and length do not typically affect cell viability^25^, but can induce reversible stress responses^26^. Consistent with this, examination of a broad number of eukaryotic cell types including embryonic stem cells demonstrate that exposure to pressures of ≤ 100 MPa for periods of 10 min or less does not significantly alter cell viability^9,27^. Melanocytes, adipose stem cells, dermal fibroblasts, keratinocytes and malignant melanoma cells similarly demonstrate no significant growth alterations upon return to atmospheric pressure after 2 min at 150 MPa^28^. Even among sensitive populations such as mammalian gametes and stem cells, exposure to 20–80 MPa for 30–120 min resulted in no significant loss of cell viability^17,29^. Rather, exposure of spermatozoa, oocytes, murine embryos, and embryonic stem cells to 60 MPa for 30 min resulted in *enhanced* viability with respect to cryopreservation, fertilizing ability, developmental competence, and differentiation^29^. In the case of mouse embryos and blastocysts, there is evidence this cryopreservation protection is likely due to the induction of stress response proteins^30^. Similarly re-expansion rates following vitrification of bovine blastocysts and porcine mesenchymal stem cells have also been shown to be significantly higher following 1 h pressure treatment in the range of 40–60 MPa compared to controls^31,32^. Such results demonstrate that even very sensitive eukaryotic cells (mammalian embryos and blastocysts, mesenchymal and embryonic stem cells) can withstand (and thrive at) pressures of 60–80 MPa for periods of 10 min or more without irreversible inhibition of cell function.

Initial experiments explored single pressure drops ranging from near atmospheric levels up to levels at which treated cells were no longer viable, in combination with several concentrations of a DNA vector successfully employed in other biophysical methods of transfection to investigate the potential of pressure mediated transfection. In the present study, we describe a rapid (< 3 min) method of cell transfection achieved via an increase in hydrostatic pressure (to 60–80 MPa), followed by a sudden decrease which we term Pressure-jump-poration (PJP). This sudden pressure decrease induces reproducible and reversible interruptions of cell membranes capable of ectopically modifying cellular properties in both primary and transformed cells. The use of such hydrostatic pressure alterations presents many ideal features for such cell modifications including: low cost per unit, high levels of biological safety (due to surety of sample-to-sample cell sterilization via high pressure cycling), efficiency relative to existing methods, lack of required additional consumables, and scalability to any cell number or volume even for difficult to transfect cell populations. This approach also allows for direct utilization of the widest possible array of existing genetic resources without need for additional modification.

## Results

**Figure 1:**
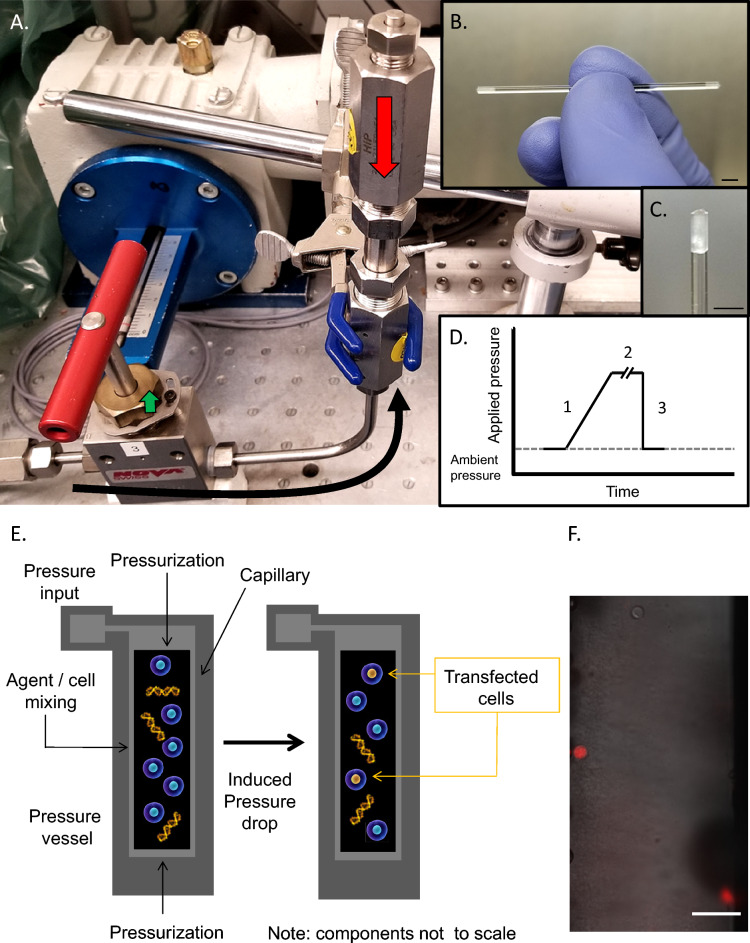
Hydrostatic pressure application to mammalian cells. (**A**) High hydrostatic pressure generating device used for experimental pressures of 1–200 MPa. Pressure line with valve seat (black arrow) and pressure chamber (red arrow) are indicated. Total chamber capacity 10 mL. (**B**) Example of cell suspension isolated in borosilicate capillary. (**C**) Closeup of capillary petrolatum seal. Scale bars in figures (**B**) and (**C**) equal to 2 mm. (**D**) Schematic of the Pressure Jump-Poration. Phase 1: Sample is pressurized from ambient to the desired pressure at a rate of ~ 65 MPa/min; Phase 2: pressure is maintained at the desired static pressure for the indicated period (0.5–10 min); Phase 3: sample pressure is suddenly returned to ambient by opening valve. (**E**) Schematic of pressure-induced transfection of genetic material. (**F**) Detection of propidium iodine uptake in select cells at 60 MPa (borosilicate capillary) immediately following acute depressurization. Scale bar denotes 100 μm.

Certain cell types, particularly transformed lines, can be efficiently transfected using several physical methods including electroporation, lipofection, and calcium phosphate-mediated uptake. In contrast, many primary cell types including embryonic stem cells are more difficult to transfect. The use of dynamic hydrostatic pressure for transfection overcomes these difficulties. Briefly, in the method we describe here (Fig. 1A–C), borosilicate capillary segments were prepared, sterilized, and filled with single cell solutions in electroporation buffer whereupon they were sealed with sterilized petrolatum to allow rapid transmission of local hydrostatic pressure (see “Methods”). Capillaries were then placed into a sealed silicone oil-filled pressure vessel. Hydrostatic pressure was then increased to the desired static holding pressure at a rate of approximately 65 MPa/min and maintained for periods of 30–600 s, whereupon this pressure was slowly or abruptly returned to ambient pressure (Fig. 1D).

**Figure 2:**
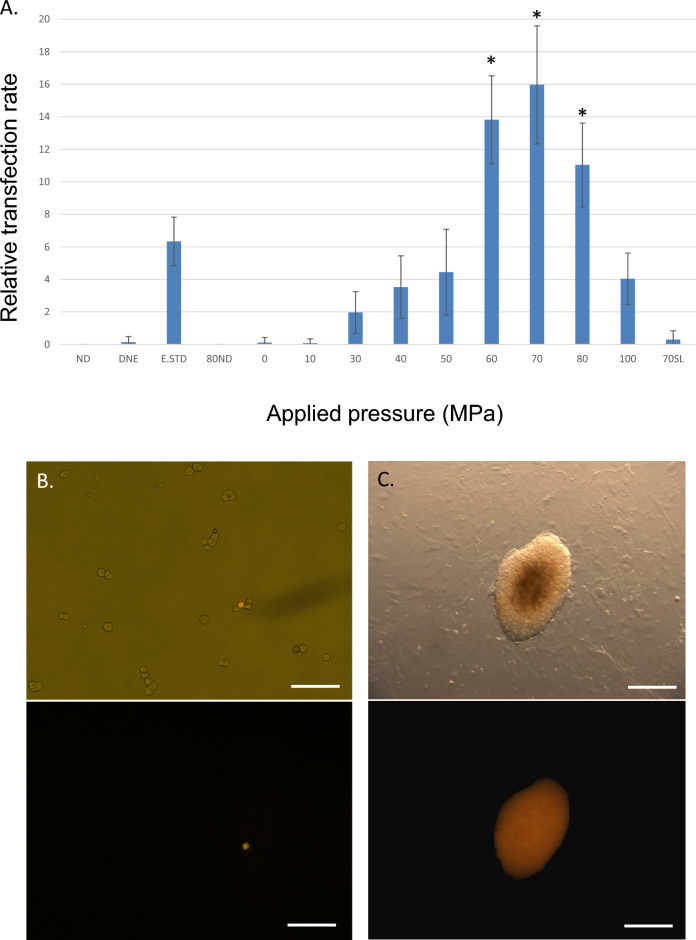
Efficacy of transfection of ES cells using pressure-jump-poration. (**A**) Transfection rate for the pressures indicated compared to standard electroporation conditions following transfection. NDN: (−) DNA control; DNE: (+) DNA, no electroporation; E.STD: (+) DNA, (+) electroporation; ND80: (−) DNA at P = 80 MPa; 0–100: (+) DNA, (+) pressure treatment; 70SL: (+) DNA, (+) pressure treatment, but with slow (3 min) pressure release. All other samples were held at the indicated pressure for 30 s followed by acute depressurization. For each pressure condition *n* = 9 independent experiments with three replicates within each experiment were performed, and *n* = 5 independent experiments with three replicates within each experiment for electroporation conditions. Results shown ± SD. (**B**) ES cells on gelatin at 24 h following pressure treatment at 60 MPa (no selection). Scale bar denotes 150 μm. (**C**) Clonal ES cell colony on fibroblast bed layer following pressure treatment at 60 MPa with subsequent puromycin selection for 5 days. All pressurization experiments were performed at a concentration of 4000 cells/μL with treated cells plated at 10,000 cells/well in a 6 well plate; electroporation standards were plated similarly. Scale bar denotes 200 μm. Results shown ± SD. *Denotes significant enhancement at *p* < 0.01 over electroporation. Transfection plasmid shown in (**B**) and (**C**) expresses dTomato/puromycin.

It was observed that sudden depressurization from a particular range of static holding pressures resulted in a small number of cells permeabilizing their outer cell membrane without overt cellular destruction (Fig. 1E). An example of this can be seen in Fig. 1F, in which the normally cell impermeant marker propidium iodide (MW 668.4) has been added to a concentration of 0.75 μm (0.5 μg/mL) to the resuspension solution. Immediately following depressurization (< 5 min) a minority of cells could be observed allowing uptake of propidium iodide suggesting such cells might also be capable of absorbing higher molecular weight entities such as plasmid DNA. As shown in Fig. 2A, following pressure-induced transfection in the presence of a ~ 9.2 kb DNA plasmid inducing puromycin resistance (Addgene # 62988), numbers of puromycin-resistant colonies were enhanced within a specific pressure range following puromycin selection an initial plating density of 1042 cells/cm^2^ (10,000 cells/well of standard 9.6 cm^2^ 6-well TC plate). We observed that static pressures in the range of 60–80 MPa followed by sudden depressurization resulted in a marked increase in plasmid uptake. Duration at these static pressures (30, 60, 90, 120, 180, 300 or 600 s) did not appear to affect the ability of cells to take up plasmid DNA. While durations of static pressure hold outside of these durations (< 30 s or > 300 s) were not examined, they are likely to yield similar results given our previous observations. As such all further cell uptake experiments were held at a static pressure for 30 s followed by sudden depressurization. To differentiate true functional plasmid expression in viable cells from simple DNA uptake, such uptake was assessed via clonogenic assay of puromycin-resistant clones. This provides demonstration of faithful plasmid uptake together with continued cell survival, propagation and function following cell transfection. Importantly it also demonstrates that transfected plasmids were actively and accurately transcribed. Plasmid uptake was found to be significantly reduced if the static holding pressure was outside the range of 60–80 MPa (Fig. 2A).

To determine the relative efficiency of PJP, results with ES cells were compared in identical solutions and DNA concentrations to an optimized electroporation standard (E STD: Biorad 240 V, 500 μF in 4 mm cuvette). As shown in Fig. 2 the values obtained compare favorably to the current standards. No transfection was observed in the absence of added puromycin-encoding DNA in either the absence (ND) or presence (ND80) of applied hydrostatic pressure; similar to that seen for electroporation (NDE). As shown in Fig. 2, even in the presence of added plasmid DNA (20 μg/mL), very low levels of transfection are observed at static pressures below 30 MPa. In addition, at optimal pressures for transfection in ES cells, low levels of transfection were observed under conditions where the hydrostatic pressure applied was reduced slowly (70SL). Though the data presented here are for ES cells resuspended in EmbryoMax electroporation buffer, results obtained using OptiMEM or serum-free basal media such as DMEM were not significantly different demonstrating similar transfection results (data not shown).

To assess post-treatment function, unselected ES cells were plated on gelatin and visualized 24 h following treatment with static pressures equal to 60 MPa (Fig. 2B). As indicated in the figure, dTomato transfected cells were capable of appropriately transcribing and translating transfected DNA markers during this period. Similarly pressure transfected ES cells plated onto DR4 multidrug resistant mitomycin-treated fibroblasts (Fig. 2C), demonstrated continued growth characteristics similar to those seen for other forms of gene transfection (electroporation, lipofection).

**Figure 3:**
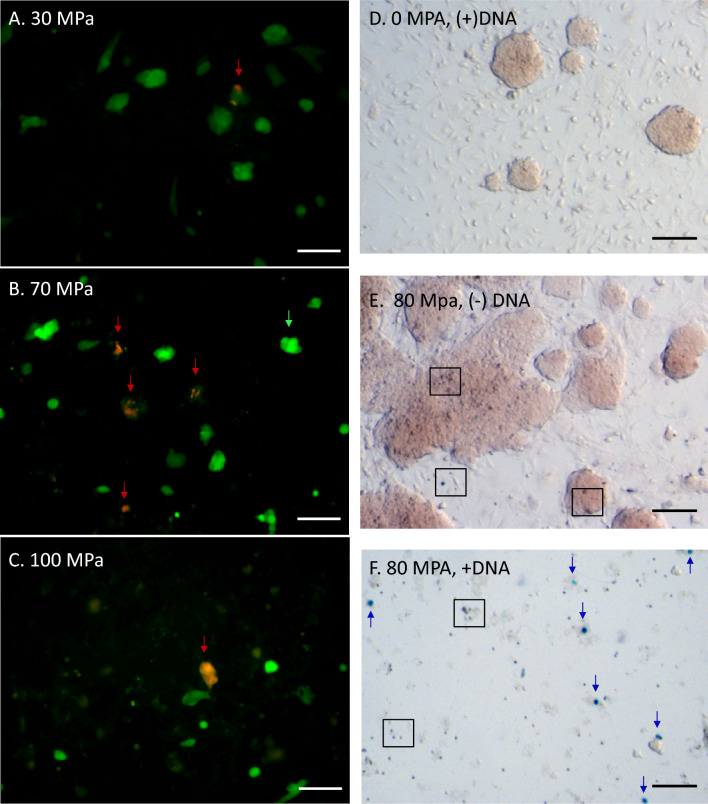
Expression of DNA constructs in mammalian cells. ES cells were transfected with plasmids containing several selection markers to assess expression competency. (**A**–**C**) Fluorescent photomicrographs of pressure treated EYFP-expressing ES cells 24 h following treatment at the pressures indicated in the absence of selection. Pressurization experiments were performed at 4000 cells/μL with cells plated at 25,000 cells/well in a 24 well plate. Relative numbers of transfected, dTomato-expressing cells are indicated (red arrow). Transfected cells exhibit reduced levels of cytoplasmic citrine compared to non-transfected cells (green arrow). Scale bar denotes 100 μm. (**D**–**F**) Beta-galactosidase expression in R1 transfected ES cells 48 h following pressure treatment. Pressurization experiments were performed at 4000 cells/μL with cells plated at 25,000 cells/well (**D**,**E**) or 10,000 cells/well (**F**) in a 24 well plate. Scale bar denotes 300 μm. (**D**) (+) DNA, (−) pressure treatment, (**E**) (−) DNA (+) 80 MPa pressure, (**F**) (+) DNA (+) pressure treatment. Beta-galactosidase positive cells are indicated (blue arrow), as are examples of dead cells following pressure treatment (box).

To further address the capabilities and characteristics of pressure transfection, experiments were conducted in a separate ES cell line expressing EYFP (citrine) from the *ROSA26* locus^33^ in the cytoplasmic cellular compartment. An advantage of this line is that it allows real-time examination for diffusion of the fluorescent 27.1 kDa EYFP protein from the cytoplasmic compartment in the event of interruption of the plasma membrane, which will recover over the long term given proper resealing of the plasma membrane. As shown in Fig. 3A–C (red arrows), transfection of EYFP-expressing ES cells using PJP at different static pressures and plated onto supporting DR4 fibroblasts (no puro selection applied) demonstrated competent expression of dTomato from the 9.5 kb plasmid vector in a minority of cells within 24 h post-transfection.

Consistent with these findings, the data in Fig. 2A show that the frequency of these events followed a general trend with respect to applied static hydrostatic pressure, with a reduction in the average intensity of EYFP expression within dTomato^+^ versus dTomato^-^ cell populations (Fig. 3B). As shown in Fig. 3D–F, similar experiments performed in ES R1 cells using a 12.3 kb Lac-Z expression plasmid demonstrated beta-galactosidase expression at 48 h following pressure treatment. Even plating at 2.5 fold higher concentrations (25,000 cells/well in a 1.9 cm^2^ 24 well plate, Fig. 3D,E) failed to demonstrate any beta-galactosidase expressing cells in the absence of either pressure treatment + DNA (Fig. 3D), or pressure treatment minus added plasmid DNA (Fig. 3E). By contrast beta-galactosidase positive cells (blue arrows) could readily be observed even at 2.5 fold lower plating densities under appropriate conditions of pressure treatment + vector (Fig. 3F). As expected, pressure treatment did result in the induction of cell death within a subpopulation of cells (boxed). Thus, using two distinct embryonic stem cell lines and several different expression vectors we observed that upon sudden depressurization after maintaining appropriate levels of static pressure resulted in the uptake and expression of ectopic DNA in a subpopulation of surviving cells.

**Figure 4:**
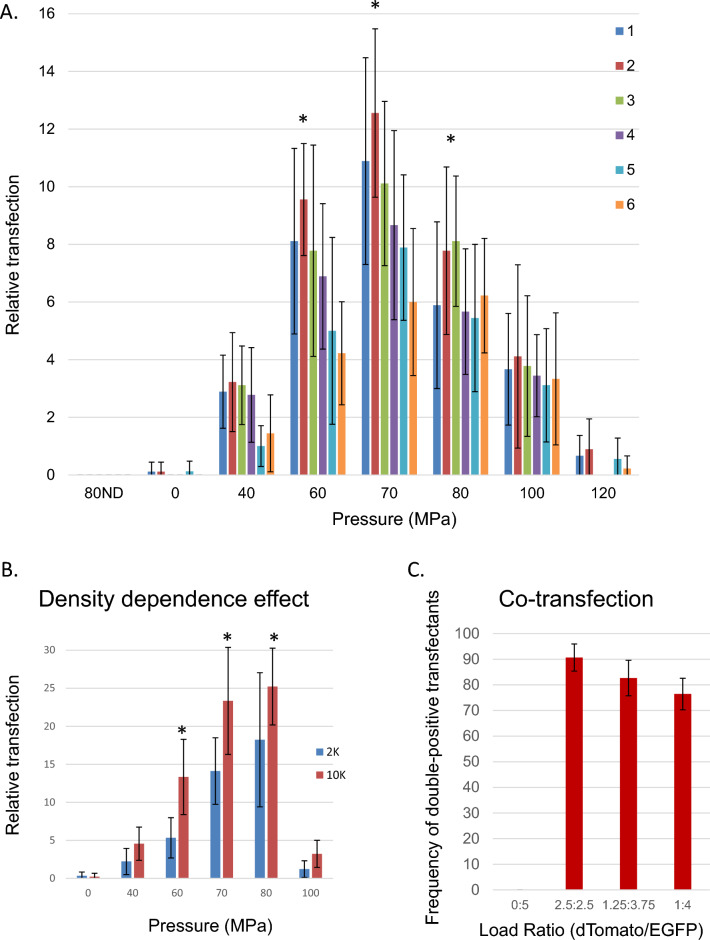
Characteristics of pressure-induced DNA transfection. All pressurization experiments were performed at a concentration of 4000 cells/μL. (**A**) Transfection response profiles of six ES cell lines to pressure treatment with treated cells plated in a 6 well plate at a density of 10,000 cells/well. *Denotes significant enhancement at *p* < 0.01 over values seen at 100 MPa. Lines: 1—R1, 2—Citrine, 3—Casp3KO, 4—RipK1KO, 5—RIPK3KO, 6—Casp8KO. (**B**) Initial plating density dependence on numbers of resulting transformants in pressure-treated ES cells. Cells were plated in a 24 well plate at 2000 or 10,000 cells/well as indicated. *Denotes significant enhancement at *p* < 0.01 over values seen at 2000 cells/well. (**C**) Co-transfection incidence in transfected R1 ES cells treated at 70 MPa with different reporter plasmid ratios at 48 h post transfection: 0 μg dTomato:5 μg EGFP; 2.5 μg dTomato:2.5 μg EGFP; 3.75 μg EGFP:1.25 μg dTomato; 4 μg EGFP:1 μg dTomato. Cells were plated at 25,000 cells/well in a 24 well plate. For each pressure condition, *n* = 3 independent experiments with three replicates within each experiment were performed. Results shown ± SD.

In order to better understand the features of this pressure responsivity in mammalian cells, parental and sublines of R1 or citrine-expressing ES cells were examined for their DNA transfection potential as a function of pressure as shown in Fig. 4A. While absolute differences in the pattern and level of DNA uptake were noted between lines, a similar pattern of responsivity across different primary cell lines was observed for static pressures between 60–80 MPa.

As with other biophysical methods of cell transfection, successful uptake of ectopic molecules represents a balance between sufficient and excessive injury to the cell membrane. Integral to this is the level of support enabling injured cells to recover. Compared to other types of primary cells, stem cells exhibit strong density-dependent effects with respect to survival and growth, therefore transfection efficiency was examined as a function of cell plating density^34^. As shown in Fig. 4B, higher initial plating densities resulted in significantly higher apparent transfection efficiency compared to lower plating densities over the pressure range of 60–80 MPa. This effect is likely due to the higher level of autocrine support present at higher cell densities in ES cells, in turn promoting better survival of transfected ES cells; rather than a direct effect on transfection efficiency.

A common feature of transfection methods which act through interruption of the cell membrane is the creation or ‘poration’ of transient holes in the phospholipid bilayer, followed by resealing on the time scale of seconds to minutes whereupon the cell membrane recovers its intrinsic impermeability^35^. To assess the ability of PJP to allow multiple plasmid uptake, the ability of pressure treated R1 ES cells to uptake multiple plasmid reporters was examined. As shown in Fig. 4C, once cells are in a state to take up one plasmid, their propensity to take up an additional reporter appears to be high, as variance of the relative reporter within the ratios indicated did little to alter the overall rate of double positive cells. This suggests that the primary limitation to transfection lies in achieving the competent uptake state, and that once achieved, the odds of continued survival with further uptake remain high, at least on the time scale of plasmid uptake.

Despite the strong propensity of ES cells to uptake multiple plasmids upon being rendered labile following PJP; the overall efficiency of this process (like many biophysical forms of gene transfection) is relatively low (< 1/100 cells). In order to better understand the population dynamics with respect to cell permeabilization following PJP, ES cells were examined at three stages, before, immediately after PJP or 15 min after PJP. As shown in Supplemental Fig. 1A, ES cells were first incubated in 10 μM Calcein-AM (green) and 5.7 μM (2 μg/mL) DAPI (blue) for 20 min at 37 °C and the resulting Calcein-AM and DAPI positive populations quantified. Immediately following pressure jump transfection at 70 MPa the populations shown in ‘Stage 2’ were characterized. Cells in which pressure treatment does not induce membrane permeabilization remain Calcein-AM+/DAPI− (approximately 59% of cells following 1 pressure jump), while the cells undergoing permeabilization of their cell membrane become DAPI+ /Calcein-AM− due to leakage in the immediate post-treatment period. DAPI positive cells present prior to treatment are indistinguishable from pressure-jump permeabilized cells by these measures. As shown in the figure, this population (DAPI+) rises from ~ 2 to ~ 41% following pressure treatment. However following the incubation of cells at room temperature for 15 min a small number of DAPI+ cells (thus disrupted by the pressure jump process), regain their membrane integrity following this recovery period as shown by their impermeability to 3 μM (2 μg/mL) propidium iodide added following recovery. Examples of this can be seen (white circles) in the population shown in Supplemental Fig. 1B. A subgroup of this population (green circles) even appears to recover some degree of Calcein-AM signal following membrane closure over this period, albeit at greatly reduced levels compared to non-disrupted cells.

**Figure 5:**
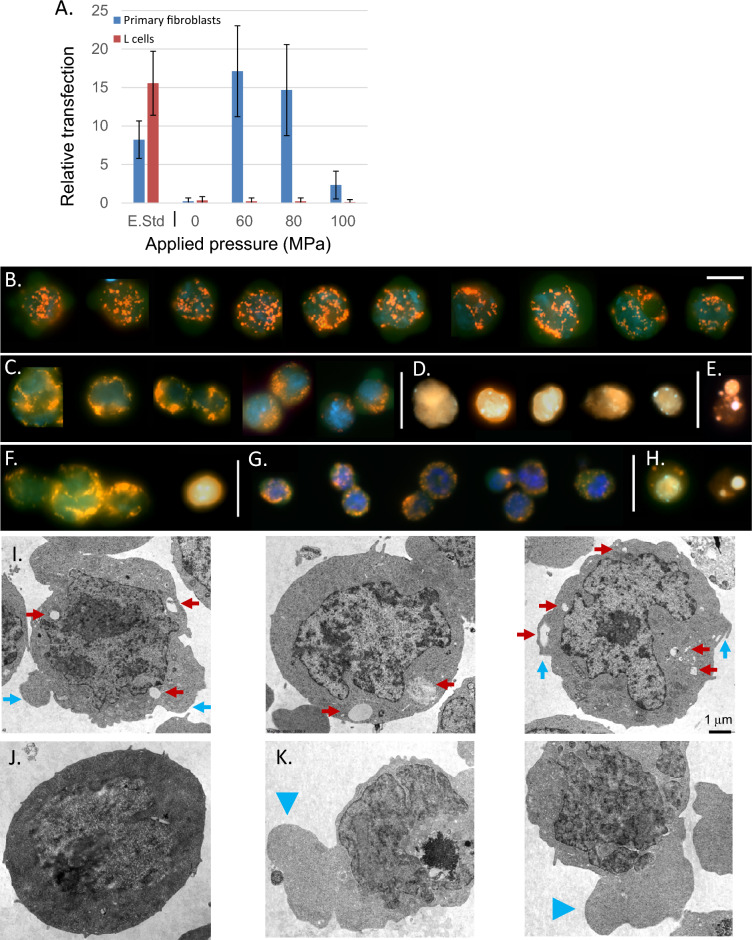
Properties of pressure-treated cells. (**A**) Differences in pressure-mediated transfection efficiency in immortalized versus primary cells. Shown are relative transfection efficiencies of primary fibroblasts (blue) vs. mouse L-cells (red) by electroporation and pressure-mediated transfection. Pressurization was performed at of 4000 cells/μL with cells plated at 10,000 cells/well in a 6 well plate; electroporation standards plated similarly. For each pressure condition, *n* = 3 independent experiments with three replicates within each experiment were performed. Results shown ± SD. (**B**–**H**) Cellular features and morphology of pressure-treated R1 ES cells. Live citrine-expressing ES cells were treated with 1.8 μM (1 μg/mL) Hoechst 33342, 10 nM (5 ng/mL) TMRM and 0.75 μM (0.5 μg/mL) propidium iodide prior to pressure treatment. (**B**) Cells in the absence of pressure treatment; (**C**) Typical appearances of ES cells following 1 min of pressure treatment at 100 MPa (pressurization at 4000 cells/μL, cells were held for 1 h at 25,000 cells/well in the slide chamber). (**D**) A small subgroup of these cells become PI+ but retain cellular features. (**E**) A major portion of these PI+ cells go on to exhibit features of cellular destruction in the immediate (30 min) post-treatment period. (**F**–**H**) ES cells following 5 min at 100 MPa. The majority of these cells demonstrate features shown in (**F**,**G**). (**G**) A portion of recovered cells exhibit features of reduced cellular volume. (**H**) The great majority of cell which become PI+ following treatment at 100 MPa for 5 min exhibit features of cellular degeneration. For (**B**–**H**) scale bar indicate in (**B**) represents 10 μm. (**I**–**K**) Electron photomicrographs of ES cells. (**I**) Following rapid depressurization at 80 MPa, a population of ES demonstrated the presence of intracellular voids proximal to the cell membrane (red arrows), frequently associated with protuberances of the cell membrane (blue arrows). ES cell held at ambient pressure is shown for comparison (**J**). By contrast ES cells subjected to slow pressure release at 80 MPa, (**K**) often demonstrated extensive extrusions (blue arrowheads). For figures (**I**–**K**) scale bar represents distance of 1 μm.

Given that several straightforward methods currently exist to efficiently transfect immortalized cell lines, we instead initially focused our efforts on more difficult to transfect cells such as ES cells. Following determination of the basic functional parameters regulating transfection of ES cells using dynamic pressure, we sought to examine the ability of PJP to transfect other cell types. Initially we anticipated the response pattern across cell types to be similar to other biophysical methods such as electroporation. However, as shown in Fig. 5A, although PJP-induced transfection of primary fibroblasts demonstrated a pattern similar to that seen for ES cells, attempts at transfection in mouse L-cells using PJP were unsuccessful. Confirmation that this effect was unrelated to the reporter plasmid utilized is demonstrated by successful transfection of both L-cells and primary fibroblasts using standard electroporation. Similar attempts to pressure transfect well-characterized immortalized cell lines HEK293T and Cos-7 (lines efficiently transfected by electroporation, calcium phosphate mediated transfection and lipofection) were similarly unsuccessful (data not shown).

While previously there has been extensive characterization of several cell types in response applied hydrostatic pressure, the current study differs in that high pressure treatment terminates in a sudden drop to ambient pressure. In order to better understand how this process affects cellular dynamics, several morphological and intracellular features were examined at the upper limit of pressure for transfection and ES cell viability. For these experiments we used citrine-tagged ES cells expressing monomeric EYFP from the *Rosa26* locus^33^, which were incubated with Hoechst 33342, tetramethylrhodamine methyl ester (TMRM) and propidium iodide at final concentrations of 1.8 μM, 10 nM and 0.75 μM respectively. As shown in Fig. 5B, cells untreated by pressure transfection exhibited intact nuclei with granules of TMRM fluorescence, indicative of dye accumulation in active mitochondria with an intact membrane potential. In Fig. 5C, the samples were held at a static pressure of 100 MPa for 1 min followed by sudden depressurization. The ES cells continued to demonstrate TMRM fluorescence, however with some reduction in the number of distinct granules suggestive of mitochondrial fusion. In addition, a small number of cells (~ 1%) began to demonstrate features indicative of entry of the cell permeability marker propidium iodide to identify membrane disruption (Fig. 5D). Additionally, a significant number (~ 30%) of cells developed morphologies like those shown in Fig. 5E over 5–30 min post-treatment. Such cells presented with overt morphologic cell disruption with membrane-bound cell fragmentation reminiscent of apoptotic cell death. If the static pressure is maintained for longer periods (5 min at 100 MPa, Fig. 5F–H), then the majority of cells continue to present intact plasma and nuclear membranes, with cytoplasmic compartments exhibiting granules of TMRM fluorescence albeit with a small decrease in the average signal intensity. A fraction of these cells (Fig. 5G) exhibited features consistent with significant reduction in cellular volume. Extended pressure treatment resulted in an increased number of cells exhibiting overt features of cellular degeneration with very few structurally intact cells exhibiting propidium iodide entry (Fig. 5H).

Given the apparent reduction in cellular volume seen under conditions of PJP, cell size was examined, and volumes estimated based upon area measurements through the cell diameter from free-floating cells as determined by optical sectioning. For ES cells, we calculated an interpolated volume of 1536.4 ± 19 μm^3^ prior to the pressure cycling process and 1250.6 ± 24 μm^3^ after exposure to 100 MPa for 5 min followed by sudden depressurization (*n* > 60). This result is consistent with an average reduction of volume of 18.6% following pressure treatment. However many immortalized lines demonstrate little change in volume, for example, HEK293T cells revealed interpolated volumes of 2022 ± 22 μm^3^ prior to pressure cycling, and 2251 ± 19 μm^3^ following exposure to 100 MPa for 5 min followed by sudden depressurization (*n* > 40); an apparent increase of 10.2%. Thus, the volume change appears to depend on the cell type.

To examine in greater detail the structural features resulting from PJP, TEM imaging was performed on ES cells fixed 5 min following exposure to one of three treatments: (1) non-pressurized cells resuspended in EmbryoMax electroporation buffer (EB) and loaded into borosilicate capillaries cells in the presence of reporter DNA for 10 min; (2) ES cells resuspended in EB loaded into capillaries in the presence of reporter DNA for 10 min followed by pressurization to 80 MPa, maintained at this pressure for 1 min, followed by sudden depressurization; (3) ES cells resuspended in EB and loaded into capillaries in the presence of reporter DNA for 10 min followed by pressurization to 80 MPa, maintained at this static pressure for 1 min, followed by slow depressurization over the course of 3 min to ambient pressure. As shown in Fig. 5I, while several treated cells exhibited features of overt rupture, a sub-population of cells experiencing sudden depressurization presented with the appearance of distinct cellular voids proximal to the cell membrane (red arrows), often in conjunction with irregular protrusions of the cell membrane and associated cytoplasm (blue arrows) compared to untreated cells (Fig. 5J). Notably little disturbance of the nuclear membrane or material was observed under these conditions. Paradoxically, ES cells experiencing a slow reduction in static pressure frequently exhibited significantly greater disruption of cell constituents, with gross disruption of cytoplasmic, membrane and nuclear constituents (Fig. 5K).

**Figure 6:**
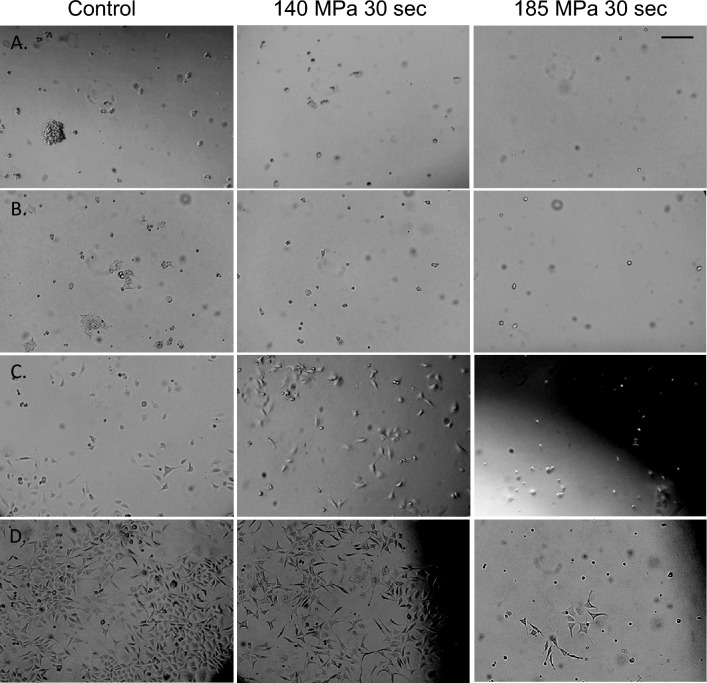
Brightfield photomicrographs of primary and transformed cells after pressure treatment. Indicated below are images of (**A**) ES R1 cells 24 h post-treatment, (**B**) HEK293T cells 24 h post-treatment, (**C**) T24 cells 24 h post-treatment, and (**D**) L-cells 72 h post-treatment for each of the conditions indicated. Scale bar in (**A**) represents 100 μm for all images in (**A**–**D**).

Based upon the functional criteria of continued survival and growth, the results demonstrated that even sensitive lines such as ES cells could survive for short periods at 100 MPa with minimal reductions in survival; however a number of transformed cell lines appear resistant to pressure-induced transfection (Fig. 5A). Given that the cellular response to pressure is known to be a function of both absolute pressure and the length of time to which cells are exposed^23,25,28^, we examined the potential nature of such differences among transfection- positive and transfection-negative lines by examining the survival properties of cell lines at different pressures and exposure periods. For each cell line investigated, equivalent cell densities were maintained at a specified pressure and time, followed by sudden return to ambient pressure. To determine relative levels of cell survival, treated cells were then plated and cultured in the manner described above. As shown in Table 1, cell types such as ES cells and primary fibroblasts demonstrated lower upper pressure limits with respect to survival compared to cell lines that exhibit transfection resistance such as mouse L-cells, exhibiting greater survival at significantly higher static pressures. A similar pattern was observed for other transfection resistant cell lines such as HEK293T, T24 and Lovo cells. Brightfield photomicrographs of these cell lines at three different levels of pressure treatment are shown in Fig. 6.

**Table 1:**
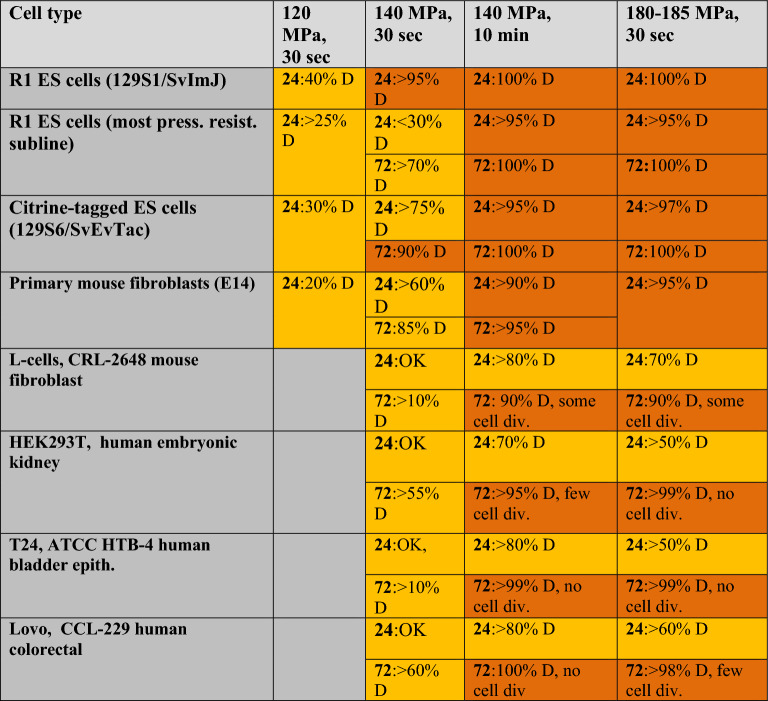
Differential survival of primary and transformed cells after pressure treatment. Pressurization was performed at a concentration of 4000 cells/μL with cells plated at 25,000 cells/well in a 6 well plate. For each of the pressure treatments listed, the average percentages of surviving cells (D = dead) were determined from the initial number plated 24 and 72 h post-treatment, 3 wells/conditions with *n* = 3 independent experiments/treatment. Results are indicated < ± 3.5%. Determinations were performed in triplicate for 3 independent replicates with general survival characteristics indicated for each of the cell line.

## Discussion

High hydrostatic pressure is successfully employed as a means of sterilizing sensitive foodstuffs in order to minimize alterations in flavor, body, texture, and nutrients^1,3^. More recently it has been used to decellularize biological materials for which traditional methods would be inappropriate and to generate peptides for anti-tumor vaccines^1,4–6,9,36^. In the current study, we have examined the use of sudden depressurization from pressures in the range of 60–100 MPa to induce gene transfection in difficult to transfect primary cells; we have termed this method Pressure-jump-poration (PJP). This method provides a quick, economical means of producing a wide range of cell transfections at any scale using DNA-based plasmids, pharmaceuticals, or other transfection agents without need for further modification. In addition, the transfection takes place in an environment which ensures sample-to-sample sterility via the insertion of high pressure (sterilizing) interphases. The results demonstrate that transfection of large plasmids is possible using this method in sensitive cell types such as embryonic stem cells. Similar to other biophysical methods such as electroporation, this transfection method does not irreversibly alter cell function of successfully transduced cells as shown by their continued growth and function^37–39^. Indeed, prior work within this pressure range exposing cells, blastocytes and gametes for longer (> 5 min) periods with successful freeze/thaw recovery, growth, development and metabolic function suggests such treatments are well tolerated even in sensitive cell types^23,30,31,40,41^_._ In fact, treatment of mouse blastocysts at 60 MPa for 30 min significantly improved their survival rates following traditional freezing and/or suboptimal culture conditions^30,31^. Thus, to investigate pressure induced transfection, initial experiments began by utilizing single pressure drops ranging from near atmospheric to the point of inducing cell death in combination with several DNA vector concentrations employed in other biophysical transfection methods. Following the observance of pressure-mediated transfection under a subgroup (pressure range) of these conditions, additional parameters were investigated to determine their effect on efficiency including the form of pressure drop (immediate versus slowed pressure release), cycles of pressure drop (1, 2, 3, etc. pressure drops on transfection efficiency), and time at static pressure (30 s to 10 min) as a function of cell type as indicated. For the current study, the most critical factor in regulating successful transfection likely lies in the fraction of cells capable of recovering from the acute injury induced by a sudden drop in pressure. In this way, the method we report here shares properties with other biophysical transfection approaches such as electroporation^35^.

In accordance with Le Châtelier’s principle, increasing pressure appears to induce cells to adapt to a smaller hydrodynamic volume^11,42^. With respect to the cellular transfection observed, such pressure-induced effects may act through the alteration of existing structural voids, particularly at sites where different biomolecular isotypes interdigitate; such as the integral membrane proteins and membrane phospholipids^20,43^. Indeed the plasma membrane appears to be a key target of pressure-induced modification over the range of pressures examined, with increasing hydrostatic pressure promoting reduction in membrane fluidity via liquid crystal to gel transition of the lipid membrane^10,20,21,43^. One intriguing element of PJP compared to other biophysical transfection methods is the differential transfection potential seen between primary versus immortalized cells. In most such methods, transfection of primary cells is significantly more difficult compared to immortalized cell lines such as Hela and L-cells^44,45^. However, using PJP transfection efficiency was significantly enhanced in primary cells such as ES cells compared to other methods such as electroporation, yet was not observed in the immortalized cell lines examined.

These results stand in striking contrast to prior observations obtained using a variety of biophysical transfection methods, suggesting differences in aspects of their underlying mechanism. Given that the differential transfection effects observed in primary versus immortalized cell lines examined may be related to their differential survival properties, we examined the response of these cell lines to applied pressure. In most cases we observed only modestly enhanced pressure resistance for immortalized versus primary cells, similar to the findings of previous studies^27,46^. These effects may reflect differences between primary and immortalized cells in the production or composition of membrane phospholipids, differential configuration of their microtubule cytoskeleton or other factors. Interestingly, cytoskeletal microtubules have been shown to be affected at the pressures examined and may play a role in disrupting the cell membrane^12–15,24,25,47^. However, the mechanism underlying the observed differences in transfection of PJP is presently unknown and remains to be investigated.

As with other biophysical methods of cell transfection, a delicate balance exists between disruptive forces promoting viable transfection and irreversible damage leading to cell death. While both electroporation and PJP are based upon permeabilizing the plasma membrane only the latter is readily scalable. This is particularly true in the case of sensitive cell types given that applied voltage increases with increasing cathode–anode gap distances. Additionally, given its known response to applied hydrostatic pressure, the risk of pressure-induced modification of genomic DNA is significantly reduced compared to potential (polyanion) damage during electroporation. While other biophysical methods such as microinjection and laser-poration also avoid such damage, these methods cannot compare in terms of speed, cost, scalability, and simplicity. Similarly, although transfection methods that use calcium phosphate, polyethyleneimine and lipid-DNA complexes have proven effective transfection strategies for a variety of cell lines (particularly transformed ones), these methods are far less effective in their application toward primary cells. Furthermore, these methods require the ongoing use of consumables, several of which (e.g., modified lipids, nucleofection) impose significant costs particularly at scale. By contrast transfection achieved by PJP requires no consumables. As a practical note, although a stepper motor-controlled pressure generator was used in the current study, the pressures employed could readily be obtained using manual devices that are commercially available.

In this initial study of the transfection of cells using sudden depressurization (PJP), the effects of pressure have been examined in isolation. Despite demonstration of increased efficiency compared to other biophysical measures there is still room for improvement. Such improvements may be realized through the addition of complimentary measures or modifications once the critical cellular interactions driving dynamic pressure transfection are better understood. For example, while reduction of cell temperature has previously been employed following electroporation to slow pore repair times, such measures were not examined in the current study.

## Materials and methods

### Reagents and cell assays

Reagents utilized include: Silicone oil (Fisher S159-500), Borosilicate glass (Harvard Apparatus, type GC150T-10, 30-0062), Petrolatum (Sigma-Aldrich, 16415), Puromycin (BioShop, PUR333.100), Geneticin (Gibco, #11811023). Plasmids used for transfections: px459V2 (Addgene # 62988), pLVX-dTomato-C1 (Takara Bio), pEGFP-puro (Addgene #45561), or pBS/KSTau/LacZ (courtesy of Dr. Friedhelm Bladt, SLRI, Mt. Sinai Hospital, Toronto), modified from TauLacZ LTNL (Dr. Peter Mombaerts, Max Planck Institute, Frankfurt). Plasmids were prepared from bacterial stocks grown overnight using EndoFree Plasmid Maxi Kit (Qiagen, #12362), with plasmid integrity verified using gel electrophoresis and DNA transfection quality independently determined using electroporation and polyethylenimine (Sigma Aldrich, 25 kDa MW, # 408727) mediated transfection of HEK 293T cells (ATCC CRL-3216) according to standard methods together with culture lines and cell media constituents as indicated below. Analyses for beta-galactosidase activity in transfected cells were performed as previously described^48^. Cell viability was assessed using standard trypan blue exclusion assay (Gibco 15250061)^49^, DAPI (Sigma D9542), propidium iodide (Sigma P4170)^50^ or Calcein-AM/ethidium homodimer live/dead assay^51^ (Molecular Probes L-3224) methods as described previously.

### Cell culture

Primary murine embryonic stem (ES) cell lines were maintained at 37 °C and 6% CO_2_ in growth media containing high glucose DMEM (Invitrogen 11960-044), 2 mM l-glutamine (Invitrogen 25030), 2 mM GlutaMAX (Invitrogen /#35050) with addition of 0.1 mM 2-mercaptoethanol (Sigma M7522), 0.1 mM MEM non-essential amino acid (Invitrogen 11140), 1 mM sodium pyruvate (Invitrogen 11360), 50 U/mL penicillin/streptomycin (Invitrogen 15140), 1000 U/mL LIF (Chemicon ESG1107) and 15% ES cell qualified fetal bovine serum (HyClone SH30071.03E). ES cells or sublines were maintained on mitomycin C inhibited multi-drug resistant mouse embryonic fibroblasts (DR4 MEFs, ATCC SCRC-1045), or on 0.1% Gelatin (Millipore ES-006-B). Additional cell lines examined in this study include: T24 (human bladder epithelial carcinoma, ATCC HTB-4) cultured in McCoy’s 5A and Lovo cells (human colorectal, ATCC CCL-229) cultured in F12 media (Gibco 11765054). HEK 293T (Human embryonic kidney, ATCC CRL-3216), Mouse L-cells (fibroblastic, ATCC CRL-2648), and primary mouse fibroblasts were all cultured in Dulbecco's Modified Eagle's Medium (DMEM) (Sigma, D5796). Each of the above media were supplemented with 10% fetal bovine serum (Invitrogen, 12483020), 2 mM glutamine (Invitrogen 25030081) and 50 U/mL penicillin/streptomycin (Invitrogen 15140). R1 ES cells (129S1/SVImJ) were generated locally as previously described^52^ and EYFP (citrine) expressing ES cells (129/SVEvTac) were a kind gift from the laboratory of Dr. M. A. Magnuson, Vanderbilt University^33^.

### Cell preparation, pressure-jump-poration, and plating

Cultured cells for hydrostatic pressure experiments were first detached from their support matrices with 0.25% Trypsin–EDTA under sterile conditions in a biosafety cabinet, centrifuged at 300×g for 4 min and gently re-suspend in EmbryoMax electroporation buffer (Millipore ES-003-D, 4.5 g/L glucose, 2.3 g/L bicarbonate, pH 7.3, 327 mOsm) or Opti-MEM (ThermoFisher 31985062, 2.5 g/L glucose, 2.4 g/L bicarbonate, pH 7.4, 285 mOsm) as indicated, with or without DNA to a final concentration of 20 μg/mL and at a cell concentration of 4 × 10^6^ cells/mL (4000 cells/μL).

Fifty-millimeter borosilicate capillary segments were pre-prepared by scoring with a diamond scribe and sterilized. Fifty microliters of cell resuspensions were then introduced to one end of a borosilicate capillary, drawing it to the far end of the tube where it was the sealed using sterilized petrolatum via syringe. Lateral displacement of cell media to the end of the tube was then performed via continued application of petrolatum, followed by petrolatum sealing on the opposing end to ensure total sample isolation from air (see Fig. 1). Enclosed capillaries were then placed into a 2-mL high-pressure microreactor (Model MS-1, HiP, Inc., Erie, PA, USA) filled with silicone oil. After closing the microreactor, the process of PJP consists of three phases: 1. pressurization, 2. maintenance of the sample at a static high pressure for a variable amount of time, and 3. sudden depressurization. In the pressurization phase, the pressure was increased to the desired level at a rate of ~ 65 MPa/min via a pressure generator driven by a stepper motor (Nova Swiss, Effretikon, Switzerland). Cells were held at the desired static pressure for periods of between 30 s to 10 min, followed by a sudden return to atmospheric (ambient) pressure. Sudden return to ambient pressure was achieved by simply opening a valve to ambient pressure. Immediately upon reaching atmospheric pressure, the pressure apparatus was disassembled and sealed capillaries were opened with a diamond scribe at which point cells were removed, counted, and plated. For the samples held for 30 s at static pressure, we estimate that the total time of experiments was 5 min. That is, following sealing the microreactor, the total time for completion of the three phases of the process and removal of the cells from borosilicate capillaries was typically 5 min.

Initially, cell pressurization experiments were also performed using sterilized heat-sealed polyethylene tubing (McMaster-Carr #5233K111). However studies were switched to borosilicate capillaries to eliminate the potential contribution of localized heat-sealing effects in altering cell membrane or solution properties. Pressure-treated cells were plated at a concentration of 2000–25,000 cells/well in standard 24-well tissue culture plates (Sarstedt 83.3922), or 10,000–20,000 cells/well in 6-well tissue culture plates (Fisher CS003516). At time points appropriate for a given analysis (immediate, 24–48 h, post antibiotic selection, etc.) numbers of transfected cells or colonies were determined as a function of the overall cell population. For immediate transfection efficiency analysis, cells were incubated with Calcein-AM and DAPI, pressure transfected and then stained with PI 15 min post-transfection. Analyses of populations were done immediately before and after pressure treatment and after PI staining. For numeric analyses/clonogenic assays of transfected cells, treated cells were allowed to recover for one day, followed by antibiotic selection for 3–5 days at concentrations predetermined to be lethal for each of the respective wild-type lines. Clonogenic assays were performed 7 days following removal of selecting agents by averaging the number of colonies in each well within a 18 × 18 mm^2^ (standard coverslip) area.

### Live cell imaging

Where indicated, pressure-treated cells were incubated with the following reagents after pressure treatment in order to monitor cellular compartments in real time: Hoechst-33342 (Life Technologies H1399, final concentration 1.8 μM or 1 μg/mL), tetramethylrhodamine methyl ester (Setareh biotech. 6275, final conc. 10 nM or 5 ng/mL, excitation wavelength (ex.) 553 nm, emission wavelength (em.) 577 nm), Propidium iodide (Sigma P4170, final conc. 0.75 μM or 0.5 μg/mL, ex. 535 nm, em. 615 nm), Calcein-AM (Invitrogen C3100MP, final conc. 5 μM, ex. 494 nm, em. 516 nm). Citrine (ex. 513 nm, em. 528 nm) was imaged on the EGFP channel.

### Microscopy

Cellular imaging was performed on a Zeiss Axio Imager.M1 microscope equipped with standard DAPI, EGFP, FITC, Rhodamine, Texas Red and Cy5 excitation/emission filters using a Marzhauser Wetzlar motorized XY controller on 40×, 63× and 100× (infinity/0.17) Plan-Apochromat and 40× Neofluar Zeiss objectives. Images were captured prior to or ~ 30 min following pressure treatments on a Hamamatsu ORCA-Flash 4.0 4MP sCMOS camera using ZEN blue 3.0 software. Images were acquired from at least five independent fields within *n* ≥ 4 separate experiments. Relative fluorescence intensity was calculated by comparing mean fluorescence intensity of cells to the background fluorescence in each field. Significance was determined by ANOVA. Images were processed using Zen software and Adobe Photoshop CS.

### Electron microscopy

Cells prior to or following pressure treatment were infused with 100 mM cacodylate buffer at pH 7.4 containing 4% para-formaldehyde and 0.1% glutaraldehyde and left overnight at 4 °C. Samples were then washed in cacodylate buffer, treated with 1% osmium tetroxide and embedded in Spurr resin as previously described^53^. Blocks were sectioned at 70 nm onto Formvar-coated 200 mesh copper grids and stained with 2% uranyl acetate and 0.1% lead citrate. Imaging was performed using a Phillips CM 100 EM microscope.

### Statistics

Statistical comparisons between individual groups were performed using Student’s *t* test (unpaired, two tailed with assumption of equal variance) for examination of significance, determined at a minimum level of *p* < 0.05. Statistical analyses of greater than two groups with one independent variable were performed using one-way ANOVA and Tukey’s post hoc with significance defined at a minimum level of *p* < 0.05. Statistical measures were performed using Microsoft Excel and GraphPad Prism Software, version 6. All values are presented as mean values ± standard deviation.

### Supplementary Information

Supplementary Figure 1.

## Data Availability

We affirm that all data generated and/or analyzed to understand and evaluate the conclusions of the paper will be archived in an approved database are available from the authors JTH and RBM. We further understand that after publication all reasonable requests for materials and data will be fulfilled within University and Tri-Council approved protocols upon reasonable request.
