# Supplementary material for: Cellular transfection using rapid decrease in hydrostatic pressure
Source: Sci Rep. 2024 Feb 26;14:4631. doi: 10.1038/s41598-024-54463-5 (PMC10897145; doi:10.1038/s41598-024-54463-5)
Supplement: Supplementary file 1 — Supplementary Figure 1. [file 41598_2024_54463_MOESM1_ESM.pdf]

Supplemental Figure 1.

A.

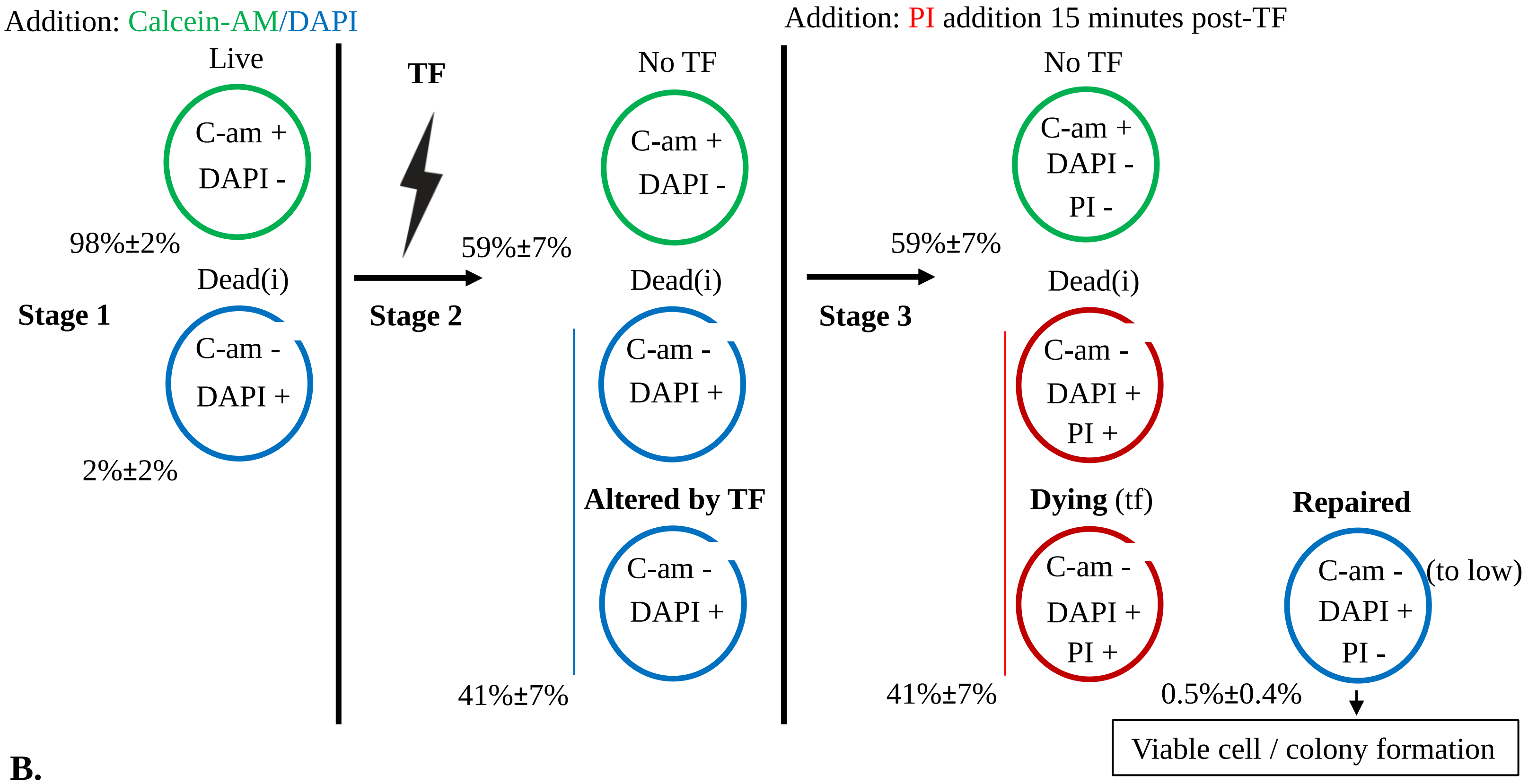

B.

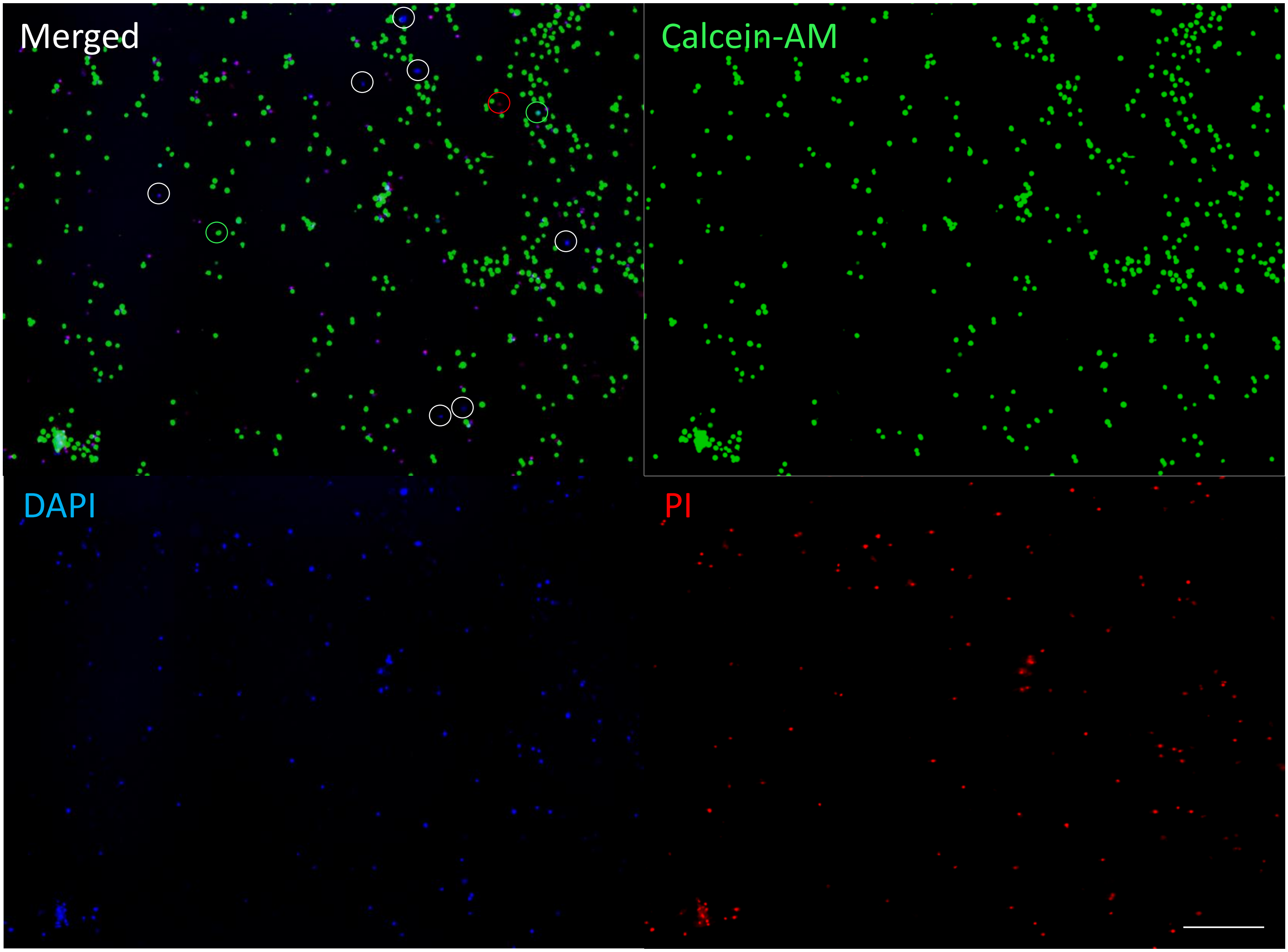

Supplemental Figure 1. Analysis of cellular states during pressure transfection. **(A)** Schematic highlighting nature and proportion of different cellular states during pressure transfection. Three stages are indicated: Stage 1 prior to transfection, Stage 2 immediately following pressure transfection, Stage 3 fifteen minutes following pressure treatment. Prior to Stage 1 ES cells were incubated in 10 μM Calcein-AM (green) and 5.7 μM (2 μg/mL) DAPI (blue) for twenty minutes at 37^0C and the resulting Calcein-AM and DAPI positive populations quantified. Pressure-jump transfection at 70 MPa immediately following this resulted in the population proportions shown in Stage 2. Cells were then incubated at room temperature for fifteen minutes at which time propidium iodide (red) was added to a final concentration of 3 μM (2 μg/mL) resulting in the fluorescent cell populations indicated in Stage 3. **(B)** Example of distribution of Stage 3 cell populations. Shown are Calcein-AM, PI and DAPI and merged channels demonstrating the relative distribution of each of the described fluorescent indicators following a fifteen minute recovery period. As shown in the Merged channel, a small population of DAPI+, PI-, Calcein-AM negative (white circle) or lowly expressing (green circle) cells can be observed. For each stage, populations were quantified from n = 4 independent experiments with 10 fields of view from each experiment. Results shown ±S.D. Scale bar equals 250 μm.
